# Imaging Findings in Patients With Benign Adenomyoepithelioma: A Retrospective Analysis

**DOI:** 10.1155/tbj/9666015

**Published:** 2026-04-24

**Authors:** Pelin Seher Öztekin, Özge Özdemir, Selen Yakıştıran, Saygın Altıner, Serap Erel, Fatma Aslan Yay

**Affiliations:** ^1^ Department of Radiology, Ankara Training and Research Hospital, University of Health Sciences, Ankara, Türkiye, akdeniz.edu.tr; ^2^ Department of General Surgery, Gazi University, Ankara, Türkiye, gazi.edu.tr; ^3^ Department of General Surgery, Ankara Training and Research Hospital, Ankara, Türkiye, ankarahastanesi.gov.tr; ^4^ Department of Pathology, Ankara Training and Research Hospital, Ankara, Türkiye, ankarahastanesi.gov.tr

**Keywords:** adenomyoepithelioma, breast, mammography, MRI, ultrasonography

## Abstract

**Background and Aim:**

Adenomyoepithelioma (AME) is a rare breast tumor characterized by the biphasic proliferation of epithelial and myoepithelial cells. Due to its rarity, data on its imaging characteristics—particularly magnetic resonance imaging (MRI) findings—remain limited. This study aimed to evaluate the radiological features of AME and assess the diagnostic contribution of multiparametric imaging.

**Methods:**

This retrospective study included 11 patients pathologically diagnosed with AME between 2013 and 2024 who underwent mammography, ultrasound (US), and MRI prior to diagnosis. Lesions were evaluated according to BI‐RADS criteria: mammography (shape, margin, density, and calcifications); US (shape, margin, orientation, echogenicity, posterior features, vascularity, and elastography); and MRI (morphology, T2 signal intensity, ADC values, and enhancement kinetics).

**Results:**

The most common mammographic finding was an isodense oval or round mass with partially obscured margins (36.4%). No lesions demonstrated irregular shape or suspicious microcalcifications. On US, 90.9% of lesions were oval/round with circumscribed (45.5%) or indistinct (54.5%) margins. All lesions were hypoechoic and vascular; 71.4% exhibited soft elasticity on strain elastography. On MRI, 54.5% were hyperintense on T2 weighted images (T2WI) with circumscribed margins. ADC values ranged from 1.00 to 1.434 × 10^−3^ mm^2^/s (mean 1.273 × 10^−3^ mm^2^/s). Enhancement kinetics demonstrated Type I in 3/11, Type II in 3/11, and Type III in 5/11 lesions.

**Conclusion:**

Although AME often demonstrates benign morphological features, it is frequently categorized as BI‐RADS 4 due to vascular and kinetic characteristics that raise suspicion for malignancy. Our findings suggest that relatively low ADC values further contribute to this suspicious imaging profile. However, the absence of suspicious microcalcifications, an oval or round shape, and hyperintensity on T2WI may aid in the differential diagnosis. Given the malignant potential of these lesions, wide local excision with negative margins is essential, and radiological findings play a crucial role in guiding the need for pathological confirmation.

## 1. Introduction

Adenomyoepithelioma (AME) is a rare biphasic breast neoplasm characterized by the proliferation of both epithelial and myoepithelial cells. First described by Hamperl in 1970, AME has historically posed diagnostic challenges due to its heterogeneous histological architecture and variable biological behavior [1]. While earlier classifications, such as Tavassoli’s, focused primarily on malignant potential [2], the current World Health Organization (WHO) classification categorizes AME as benign or malignant [3]. However, more recent proposals by Rakha et al. have introduced a more refined framework, further subcategorizing AME into benign, atypical, and malignant forms to better reflect its biological spectrum and improve consistency in reporting. In this approach, the term “malignant AME” is used to encompass malignant tumors, whether in situ or invasive, regardless of the malignant cell type. Benign AME, which constitutes the majority of reported cases and includes all lesions in our series, is characterized by the preservation of the normal bilayered epithelial–myoepithelial architecture without cytological atypia [4].

AME are frequently benign, whereas malignant forms are exceptionally rare [3, 4]. It has also been reported in the literature that they are associated with invasive ductal carcinomas and recurrences following their resection [5]. Their metastatic potential has been reported in case reports [6]. Although core needle biopsy is the first choice to diagnose AME histopathologically, it has been noted that it might yield false results [7]. The treatment of choice is surgical excision with clear surgical margins [3].

Due to the rarity of AME, the literature describing its radiological characteristics—particularly magnetic resonance imaging (MRI) findings—remains limited [5, 7–10]. In early case series, it was stated that the most common mammographic and ultrasonographic (US) finding was a noncalcified, irregularly marginated mass [9]. It was reported that AME should be considered in the differential diagnosis of solid masses with hypervascularity on US examination [9, 10]. However, more recent studies have reported that AME lesions may frequently exhibit circumscribed margins and may not display structural distortion [10]. Although conventional radiological imaging findings are described in greater detail in the literature, only limited data are available on MRI findings. In the literature, while benign criteria such as high signal intensity on T2‐weighted images (T2WI) and circumscribed margins have been reported for the MRI features of AME, it has also been stated that AME cannot be reliably distinguished from a malignant lesion due to its contrast enhancement kinetics [9, 10]. In addition, AME has been reported in a single case presenting as nonmass enhancement on MRI, further contributing to its overlap with malignant lesions [10]. AME may mimic both benign lesions (such as fibroadenoma and adenosis) and malignant lesions (such as invasive carcinoma) on imaging, making differential diagnosis challenging.

This retrospective study aimed to review the radiological findings of benign AME and to evaluate the contribution of multiparametric breast MRI—which is not sufficiently addressed in the literature due to limited data—to the differential diagnosis based on 11 pathologically proven cases.

## 2. Materials and Methods

### 2.1. Ethical Considerations

This study was reported in accordance with the Strengthening the Reporting of Observational Studies in Epidemiology (STROBE) guidelines. This study was conducted in accordance with the ethical principles of the Declaration of Helsinki. The study protocol was approved by the Institutional Ethics Committee of Ankara Training and Research Hospital (January 22, 2025; Project No: E‐25‐400). Due to the retrospective nature of the study, the requirement for informed consent was waived by the ethics committee.

### 2.2. Patient Population

The study group included 11 female patients, aged between 41 and 60 years (mean age: 52.18 years; median age: 55 years), who were admitted to our hospital between 2013 and 2024. Of these patients, 78% were in the postmenopausal period. Four patients had a family history of breast cancer, with no known genetic mutation predisposition. The study included patients who had available mammography, US, and MRI examinations, with images accessible via PACS, and who underwent core biopsy and excisional biopsy at our hospital. Patients with inadequate image quality, those who did not undergo biopsy, or those whose biopsy results were unavailable were excluded from the study.

Clinical findings: Three of the 11 patients had a palpable mass, two of the 11 had mastalgia, and the other six patients were asymptomatic. Three lesions were incidentally discovered on screening MRI, whereas eight lesions were detected on mammography and/or complementary breast US. None of the patients had a history of previous breast cancer or breast surgery.

All patients who underwent digital mammography were imaged using a full‐field IMS Giotto digital mammography system (IMS, Bologna, Italy), acquiring standard craniocaudal (CC) and mediolateral oblique (MLO) projections. Images were evaluated on a 5‐megapixel diagnostic monitor (Totoku MS 53i2, Totoku Electric Co., Tokyo, Japan).

US examinations, including gray‐scale and color Doppler imaging, were performed using a Hitachi Hi‐Vision Preirus system (Hitachi Medical Corp., Tokyo, Japan) with a 6–13 MHz, 50 mm linear transducer. Strain elastography was additionally performed in seven patients.

MRI examinations were performed using a 1.5‐T scanner (Magnetom Aera, Siemens Healthineers, Erlangen, Germany). Patients were scanned in the prone position with a dedicated 18‐channel, phased‐array, bilateral breast coil. The MRI protocol included axial T1‐weighted (T1W) and turbo inversion recovery magnitude (TIRM) sequences, axial fat‐suppressed T1 gradient‐echo sequences (precontrast and postcontrast at 1, 2, 3, 4, and 5 min), delayed postcontrast sagittal fat‐suppressed T1W images (at 6 min), and diffusion‐weighted imaging (DWI) with b‐values of 0, 400, and 800 s/mm^2^.

The morphological features of the lesions on all imaging modalities were described and evaluated according to the BI‐RADS Atlas.

On mammography, the lesions were evaluated for shape, margin, density, and the presence of calcifications; on US, their shape, margin, orientation, echogenicity, and posterior acoustic enhancement features were assessed. The longest dimension of the lesions and any other additional findings were noted (including accompanying calcification, the presence of cystic components within the lesion, elastographic evaluation, etc.). Lesion vascularity was assessed on color Doppler ultrasound (CDUS). In dynamic contrast‐enhanced MRI examinations, lesion shape, margin, T2 signal intensity, ADC values, and contrast enhancement kinetics were evaluated.

### 2.3. Pathologic Evaluation

All lesions underwent US‐guided biopsy using a 14‐gauge fully automatic needle, with at least three specimens collected. Subsequent excisional biopsies were performed following US‐guided wire localization.

Immunohistochemical analysis was performed in selected cases to confirm the biphasic epithelial–myoepithelial nature of the lesions. The myoepithelial component demonstrated positive staining for markers including smooth muscle actin (SMA) and p63, highlighting the proliferation of myoepithelial cells surrounding the epithelial elements. These findings supported the definitive diagnosis of AME.

### 2.4. Statistical Methods

This study was purely descriptive. Continuous variables are presented as mean and median values, while categorical variables are expressed as counts and percentages (*n* [%]). No comparative statistical tests were performed; therefore, *p*‐values are not reported. All analyses were conducted using IBM SPSS Statistics for Windows, Version 22.0 (IBM Corp., Armonk, NY, USA).

## 3. Results

Eight of the 11 lesions were located in the right breast and three in the left. The lesions were distributed across different quadrants, with two located in the retroareolar region. Lesion sizes ranged from 5 to 34 mm, with a mean size of 11.18 mm.

Tables 1, 2, 3 summarize the mammography, US and MRI findings of the 11 patients with AME.

**TABLE 1:** Mammography findings (*n* = 11).

Category	Subcategory	*n*: 11 (%)
NEGATIVE		5 (45.4)

MASS		6 (54.6)

Shape	Oval/round	6 (100)

Margin	Obscured (partially)	4 (66.7)
	Indistinct	2 (33.3)

Density	Iso	5 (83.3)
	Hyper	1 (16.7)

CALCIFICATION	Present	
	‐Coarse‐macro	1 (9.1)
	‐Grouped round calcifications	1 (9.1)
	Absent	9 (81.8)

COMPOSITION	Type b	3 (27.2)
	Type c	6 (54.5)
	Type d	2 (18.1)

**TABLE 2:** Ultrasonography findings (*n* = 11).

Category	Subcategory	*n* (%)
Shape	Oval	6 (54.5)
	Round	4 (36.4)
	Irregular	1 (9.1)

Margin	Circumscribed	4 (36.4)
	Not circumscribed (indistinct)	7 (63.6)

Orientation	Parallel	10 (90.9)
	Nonparallel	1 (9.1)

Echo pattern	Hypoechoic	11 (100)
Posterior features	Absent	11 (100)
Vascularization	Present	11 (100)
Elastography (in 7 lesion)	Soft Hard	5 (71.4) 2 (28.6)

BI‐RADS	4a 4b 4c	7 (63.6) 3 (27.3) 1 (9.1)

**TABLE 3:** MRI findings (*n* = 11).

Category	Subcategory	*n* (%)
Shape	Oval	6 (54.5)
	Round	4 (36.4)
	Irregular	1 (9.1)

Margin	Circumscribed	6 (54.5)
	Not circumscribed	5 (45.4)

T2 signal intensity	Hyper	6 (54.5)
	Iso	4 (36.4)
	Hypo	1 (9.1)

ADC values (10^−3 ^mm^2^/s)	< 1.5	11 (100)
Kinetic curves	Persistent	3 (27.3)
	Plateau	3 (27.3)
	Washout	5 (45.4)

BI‐RADS	4 5 6	8 (72.7) 2 (9.1) 1 (18.2)

Mammographic findings: Of the 11 patients, two (18.2%) had dense breast tissue, six (54.5%) exhibited heterogeneously dense tissue, and three (27.3%) showed a scattered fibroglandular density pattern. Six (54.5%) patients with AME had positive mammographic findings. The lesions could not be distinguished due to dense and heterogeneously dense breast patterns in 5 (45.5%) patients. The most common mammographic finding was a round or oval isodense mass with partially obscured margins due to surrounding fibroglandular tissue (36.4%). One lesion appeared as a high‐density mass with indistinct margins while another appeared as an isodense mass with indistinct margins. No lesion showed an irregular shape or margin on mammography.

None of the lesions demonstrated suspicious microcalcifications on mammography. In one case, grouped round calcifications adjacent to the lesion were identified, and the pathology of the excisional biopsy revealed an additional papilloma. Superimposed coarse and round calcifications were detected in another lesion.

US findings: 10 of the 11 lesions (90.9%) were oval or round, and one (9.1%) was irregular. Five lesions (45.5%) had circumscribed margins, while six (54.5%) had indistinct margins. Ten of the 11 lesions (90.9%) were oriented parallel, while one lesion was not. All lesions were hypoechoic and showed vascularity on CDUS. Among the seven lesions evaluated with strain elastography, five were soft, and two were hard. None of the lesions contained a cystic component.

Based on mammographic and/or breast US findings, the final BI‐RADS categories assigned were as follows: 7/11 (63.6%) lesions were classified as BI‐RADS 4a, 3/11 (27.3%) as BI‐RADS 4b, and 1/11 (9.1%) as BI‐RADS 4c. Vascularization features were identified as key factors in categorizing lesions as BI‐RADS 4a.

MRI findings: On MRI, AME lesions most commonly showed hyperintensity on T2WI (54.5%), circumscribed margins (54.5%), and a round or oval shape (90.9%). One lesion (9.1%) exhibited an irregular shape and margins.

The ADC values ranged from 1.00 × 10^−3^ mm^2^/s to 1.434 × 10^−3^ mm^2^/s (mean 1.273 × 10^−3^ mm^2^/s). Following intravenous gadolinium administration, 10 of 11 lesions (90.9%) showed rapid, homogeneous enhancement, and one (9.1%) showed rapid, heterogeneous enhancement.

In delayed dynamic series, five (45.5%) lesions exhibited washout (Type III), three (27.3%) showed plateau (Type II), and three (27.3%) demonstrated persistent (Type I) kinetic curves.

In one case, nonmass enhancement with a focal area distribution was observed accompanying the lesion. In this instance, mammographic examination revealed grouped round calcifications adjacent to the lesion, which were identified during excisional biopsy as corresponding to a benign papillary lesion.

On MRI, benign features such as hyperintensity on T2WI and circumscribed margins were observed in several lesions, although the vascular, kinetic, and relatively low ADC characteristics often mimicked malignancy. Consequently, MRI could not reliably differentiate the lesions from malignancy, with eight of the 11 (72.7%) lesions classified as BI‐RADS 4, two (18.2%) as BI‐RADS 5, and one (9.1%) initially categorized as BI‐RADS 6 due to prior discordant biopsy results.

All patients initially underwent ultrasound‐guided core biopsy. Seven lesions were correctly diagnosed as AME on biopsy, whereas four were initially misdiagnosed as nodular adenosis (*n* = 2), flat epithelial atypia (*n* = 1), and invasive ductal carcinoma (*n* = 1). Following surgical excision, all lesions were histopathologically confirmed as benign AME, with no atypical or malignant features identified. The follow‐up ranged from 6 to 120 months. None of the patients with sufficient follow‐up periods developed recurrence or additional breast malignancy.

Representative cases are presented in Figures 1, 2, 3.

FIGURE 1(a–i): A 59‐year‐old patient presenting with a palpable mass in the left breast: Digital mammography CC and MLO views of the left breast (a) demonstrate a hyperdense oval mass with indistinct margins (arrow). US shows (b) a parallel‐oriented hypoechoic oval lesion with indistinct margins, which is vascular on CDUS. Elastographic examination revealed the lesion to be hard. US‐guided biopsy yielded flat epithelial atypia. On MRI, the lesion exhibited hyperintensity on fat‐suppressed axial T2WI (c). ADC mapping revealed a mean ADC value of 1.434 (x10^−3^ mm^2^/s) (d). On the first postcontrast MIP image, rapid homogeneous contrast enhancement was observed in the lesion (e). On the late postcontrast sagittal T1WI, the lesion retained contrast enhancement (plateau) (f). The specimen radiograph of the excised lesion is shown (g). In the final excisional biopsy pathology, benign adenomyoepithelioma was diagnosed. Higher magnification demonstrates tubular structures lined by inner epithelial cells and outer myoepithelial cells, consistent with the biphasic nature of adenomyoepithelioma (H&E, x200) (h). Immunohistochemical staining for p63 highlighting the myoepithelial cell component (x100) (i).

**Figure:**
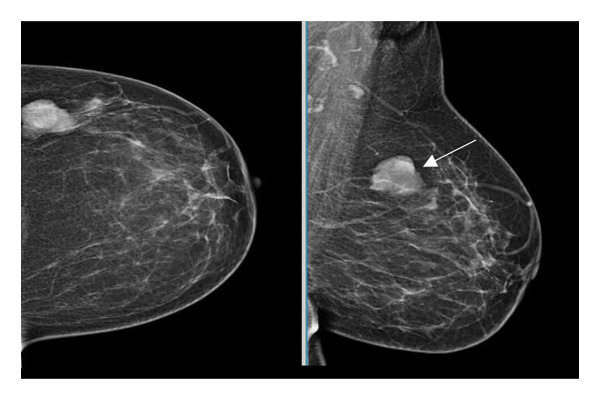
(a)

**Figure:**
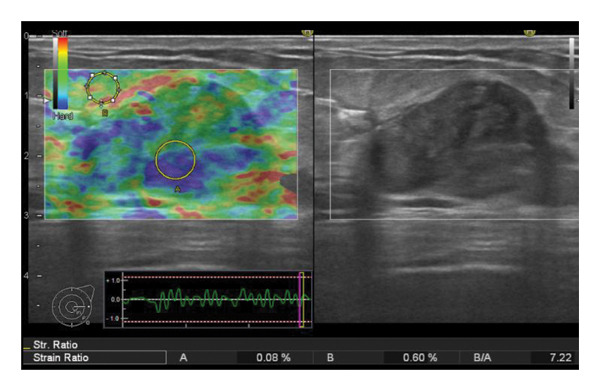
(b)

**Figure:**
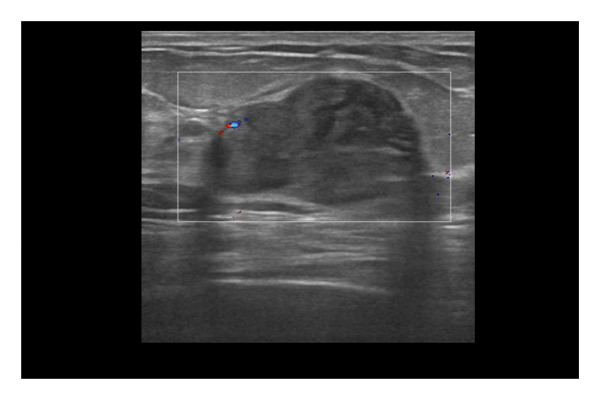
(c)

**Figure:**
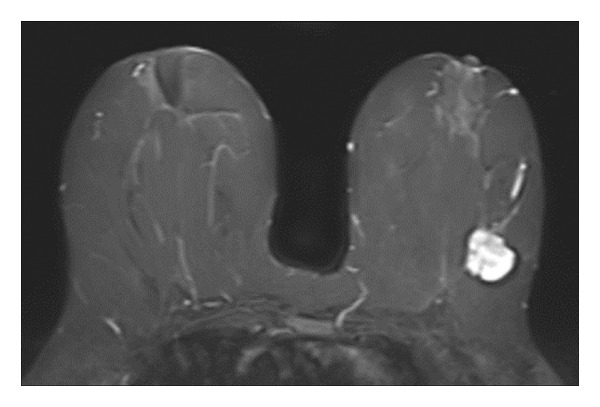
(d)

**Figure:**
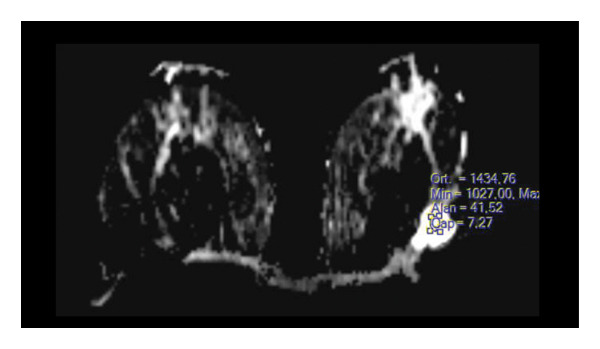
(e)

**Figure:**
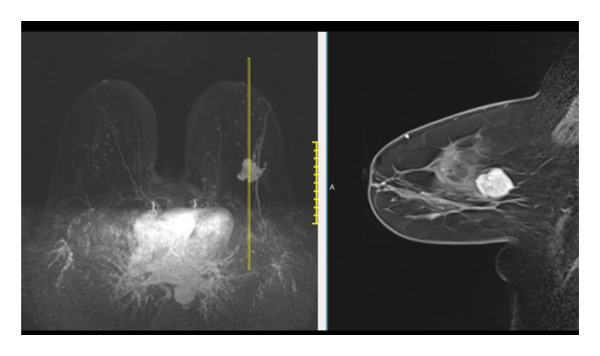
(f)

**Figure:**
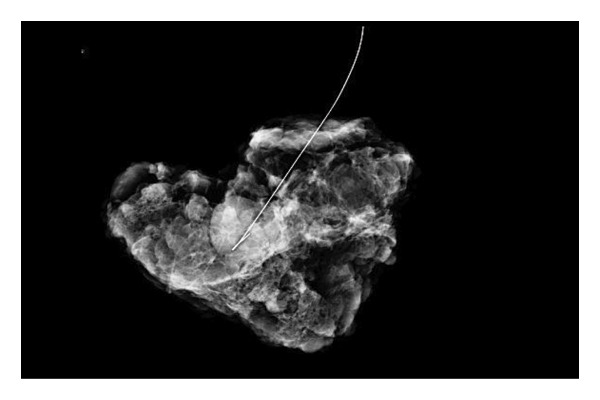
(g)

**Figure:**
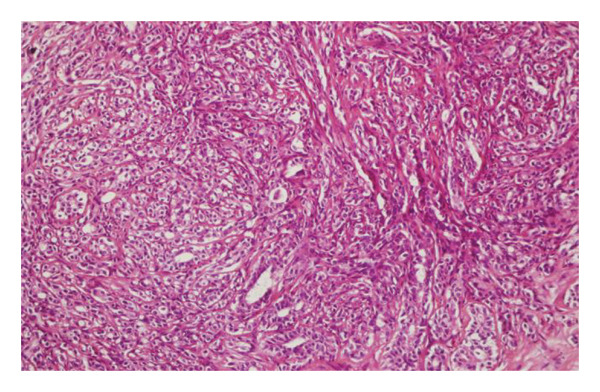
(h)

**Figure:**
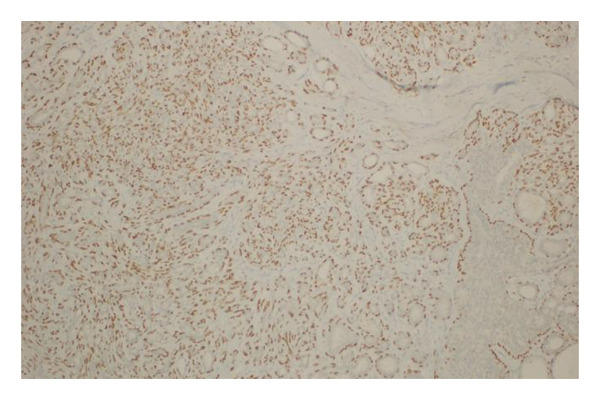
(i)

FIGURE 2(a–h): A 42‐year‐old female patient presenting with bilateral breast pain. US demonstrates a mass with nonparallel orientation, irregular shape, and indistinct margins, which is vascular on Doppler imaging (arrows) (a). Magnified specimen radiography reveals the lesion to have obscured margins and isodensity, with grouped round calcifications observed in the adjacent tissue (arrow) (b). On DWI and ADC mapping, it shows the lesion’s mean ADC value to be 1.083 (x 10^−3^ mm^2^/s) (c). On the first postcontrast MIP image, the lesion exhibits rapid, homogeneous contrast enhancement, and linear nonmass enhancement is observed posteriorly in the lesion’s vicinity (arrow) (d). Fat‐suppressed sagittal T1WI reveals an irregularly margined lesion with retained contrast enhancement (plateau) (e). Post‐excisional biopsy specimen radiography confirms the wide excision of the lesion (f). Pathology results from both US‐guided core biopsy and excisional biopsy indicate benign adenomyoepithelioma. At intermediate magnification, the tumor exhibits a biphasic architecture with glandular formations composed of epithelial cells surrounded by a prominent myoepithelial component (H&E, x200) (g). At immunohistochemical staining, S100 highlights the myoepithelial cell component (x100) (h).

**Figure:**
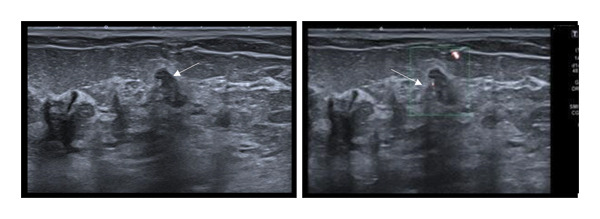
(a)

**Figure:**
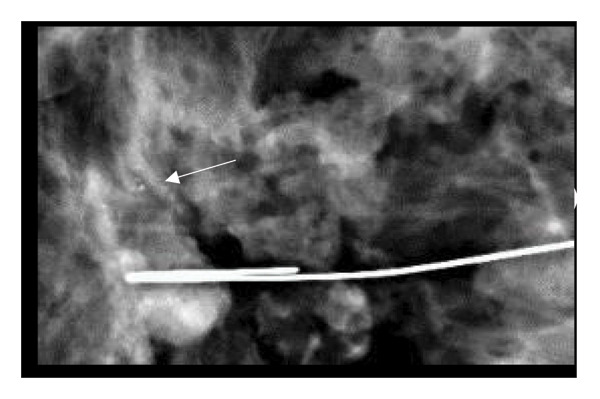
(b)

**Figure:**
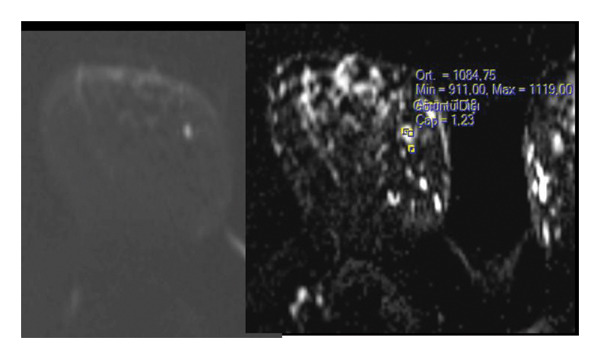
(c)

**Figure:**
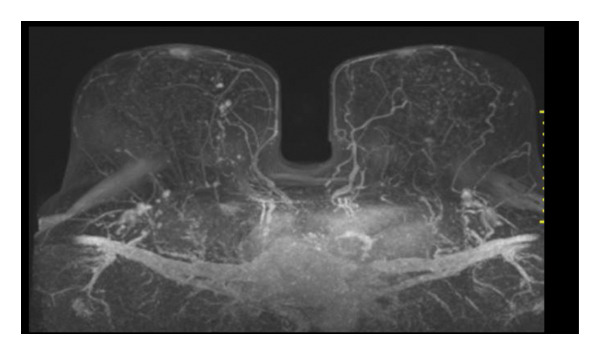
(d)

**Figure:**
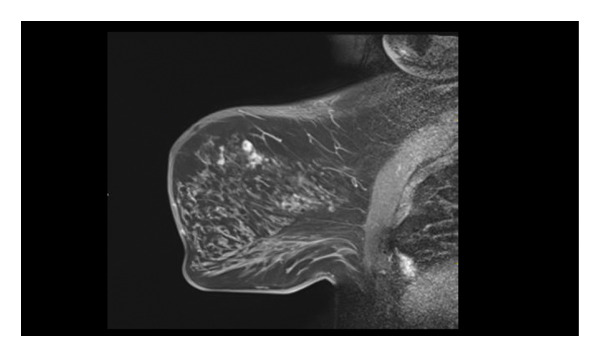
(e)

**Figure:**
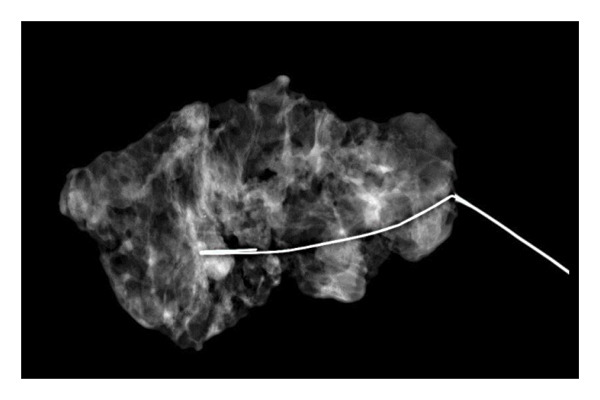
(f)

**Figure:**
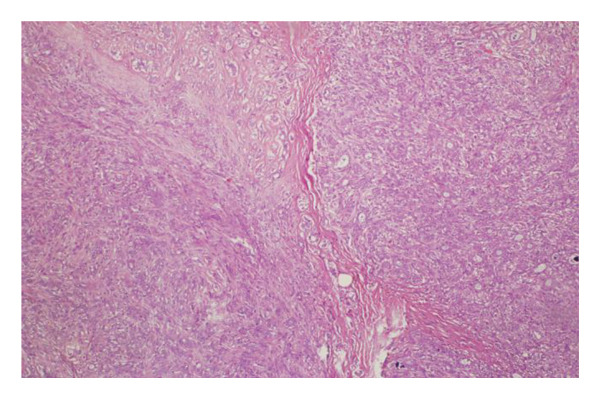
(g)

**Figure:**
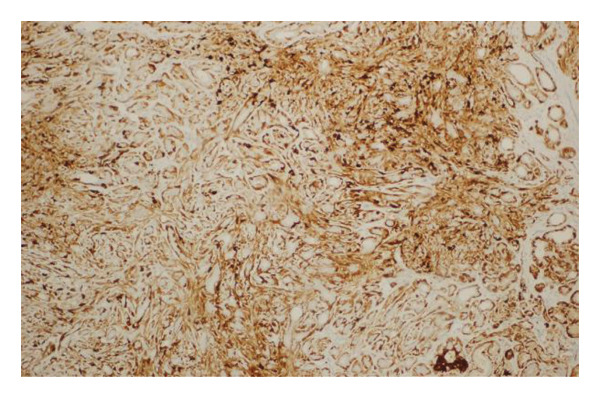
(h)

FIGURE 3(a–g): A 47‐year‐old female patient with no complaints and no findings on mammography. A mass lesion incidentally detected during screening MRI performed due to family history was further evaluated with a second‐look US for biopsy. The US examination revealed a round, well‐circumscribed mass lesion in the left breast. The lesion appeared vascular on Doppler imaging (arrow) and soft on elastography (a). On DW‐MRI, the lesion’s mean ADC value was 1.084 (x10^−3^ mm^2^/s) (b). On late postcontrast, fat‐suppressed sagittal T1WI, the lesion was well‐circumscribed and round (arrow) (c). Type III kinetic curve was obtained from the lesion (d). US‐guided biopsy and excisional biopsy revealed the pathological diagnosis of benign adenomyoepithelioma. A relatively well‐circumscribed biphasic tumor with focal pseudocapsule formation, composed of epithelial and myoepithelial elements forming predominantly tubular structures (H&E, x100) (e). A high‐power view shows myoepithelial cell proliferation without significant cytologic atypia or increased mitotic activity (H&E, x400) (f). Immunohistochemical staining for p63 demonstrates nuclear positivity in the myoepithelial cells surrounding the glandular structures (x100) (g).

**Figure:**
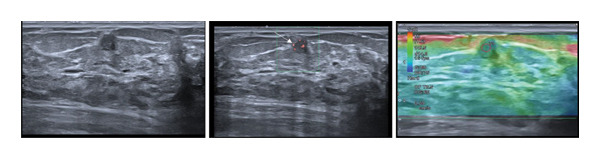
(a)

**Figure:**
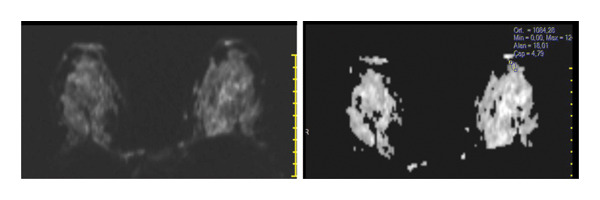
(b)

**Figure:**
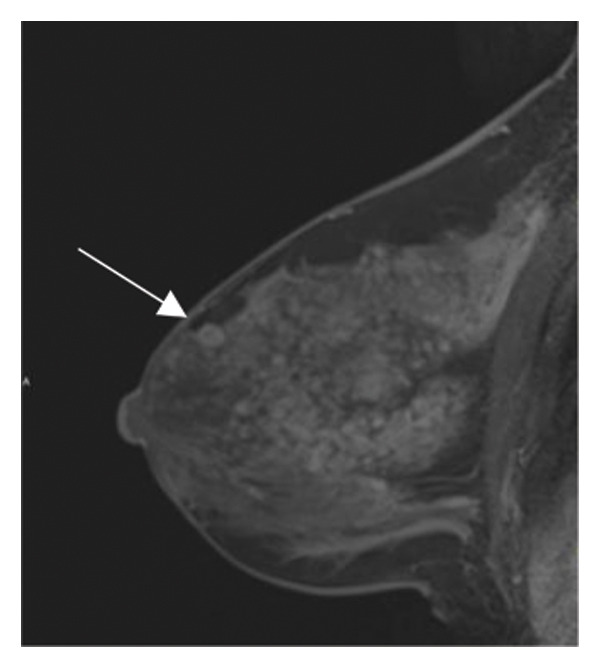
(c)

**Figure:**
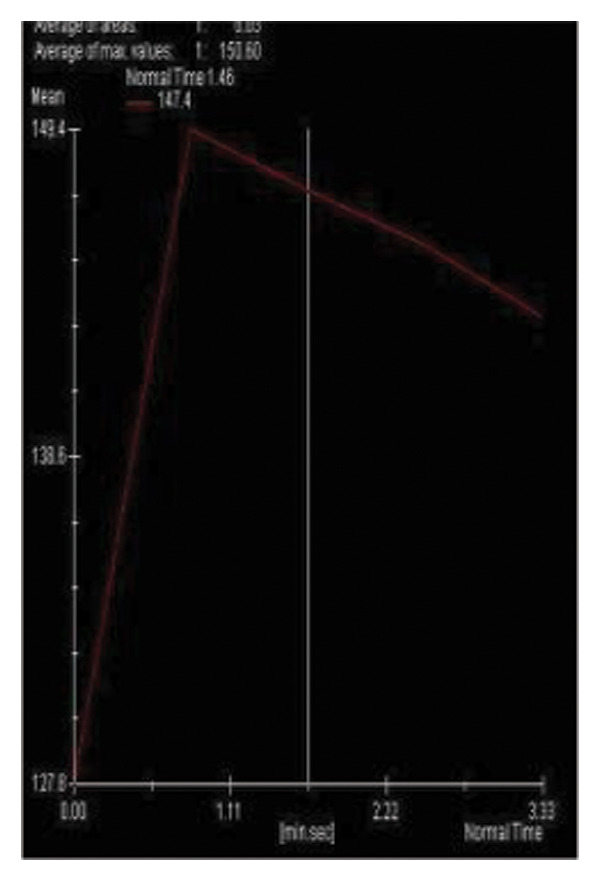
(d)

**Figure:**
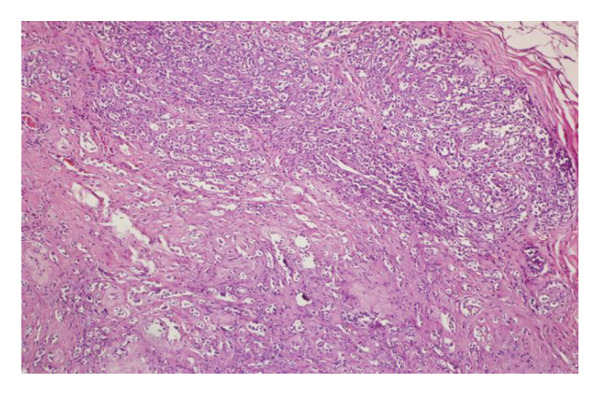
(e)

**Figure:**
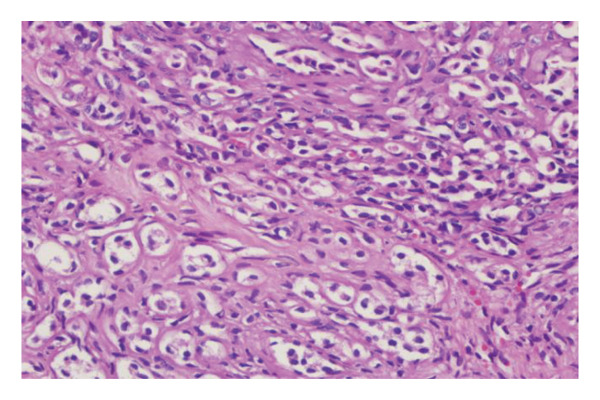
(f)

**Figure:**
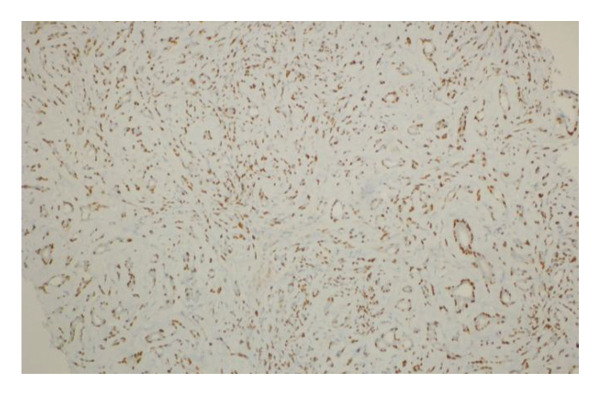
(g)

## 4. Discussion

According to the literature, AME occurs in women aged 26–81 years, with its incidence increasing with advancing age [7–9]. In their study, Zhang et al. reported a mean age of 42.2 years [11]. The median age reported in a large study published by Haque et al. in 2020, which included 110 AME cases, was 67 years [12]. In our study, 78% of the patients were older than 50 years, and the median age was 55. The size range reported in the literature is between 0.3 and 7 cm, and the average size is 2–2.5 cm [7–9, 11, 12]. Although the lesion sizes in our study were within the range reported in the literature, the mean size was smaller (11.18 mm). One reason for this is that three lesions smaller than 1 cm in our study were incidentally detected on MRI examination. Another possible explanation is that, although these lesions were small in size and exhibited benign morphology on US, they demonstrated prominent internal vascularity on CDUS. As a result, they were biopsied due to their high vascularity, leading to an earlier diagnosis. In the literature, some cases of benign AME have been reported in association with breast malignancies such as invasive ductal carcinoma and ductal carcinoma in situ [5, 8]. However, no additional breast malignancies were detected in any of the cases included in our study.

There are more data available on the mammographic and US findings of AME in the literature, and there is a relative consistency in conventional radiologic findings. Mammographic findings of AME are nonspecific, with the most common presentations being round and oval masses with well‐defined or partially obscured margins [5, 7–13]. Similarly, Smith et al. reported that AME most frequently presents as an oval mass but may also manifest as focal asymmetry or remain mammographically occult [10]. Microcalcifications within the lesion are uncommon [5, 7–13]. In our study, AME lesions predominantly demonstrated round and oval shapes with partially obscured margins, consistent with the literature. Furthermore, five lesions could not be distinguished on mammography due to dense and heterogeneously dense breast parenchyma, which aligns with the occult presentations described in recent reports [10]. Consistent with the literature, none of the lesions in our study showed suspicious calcifications.

US is often used as a complementary method to mammographic screening and for the second‐look evaluation of lesions incidentally detected on MRI. The literature states that the most common findings of AME on US examination are hypoechoic masses with circumscribed or indistinct margins and an oval or irregular shape [5, 7–13]. In our study, the lesions most commonly presented as hypoechoic masses with oval shapes and indistinct margins. Consistent with previously published studies, including Smith et al., the AME lesions in our study also demonstrated internal vascularity on CDUS [5, 9, 10, 14]. Although this finding is not sufficient for diagnosing AME, in our study, it contributed to assigning lesions with benign morphology on conventional imaging as BI‐RADS 4a and supporting the biopsy recommendation.

There are few studies or case series in the literature regarding the MRI findings of AME, and heterogeneous imaging features have been reported across studies [7–11, 13]. Previous studies have shown that AME lesions may demonstrate variable margin and shape characteristics, as well as heterogeneous internal enhancement and variable kinetic patterns, including washout (Type III) kinetic curves [7–11, 13]. In the study by Shin et al., which specifically evaluated MRI findings of benign AME lesions, the most common features were irregular margins (61%), intermediate‐to‐low signal intensity on T2WI (89%), heterogeneous internal enhancement patterns (94%), and washout kinetic curves (84%) [7]. In contrast, Zhang et al. observed that benign AME cases predominantly demonstrated persistent (Type I) kinetic curves, whereas washout (Type III) kinetic curves were more commonly associated with malignant lesions [11]. Smith et al. demonstrated in a single case that benign AME can present as clumped nonmass enhancement on breast MRI, further underscoring the significant radiologic–pathologic overlap with malignant breast lesions [10]. Additionally, Parikh et al. reported non‐enhancing internal septations in one case [8]. In our study, the most common MRI features were circumscribed margins, hyperintensity on T2WI, rapid wash‐in, and wash‐out kinetic curves on postcontrast series. None of the lesions presented as nonmass enhancement. While our findings regarding lesion morphology and T2 signal characteristics are in agreement with those reported by Zhang et al. and Smith et al., our kinetic data differ from Zhang’s benign cohort. The predominance of washout kinetic curves in our study, consistent with the findings of Shin et al. [7], further supports the observation that benign AME frequently mimics malignancy on dynamic contrast‐enhanced MRI. This kinetic profile, despite the benign nature of the lesions, supports the necessity of biopsy for a definitive diagnosis. In the present study, histopathological examination confirmed the diagnosis of benign AME in all cases, with no atypical or malignant features. The absence of cytological atypia or malignant features in our series may explain the relatively benign morphological characteristics (oval shape, circumscribed margins) observed, despite the suspicious enhancement kinetics.

There are very limited data in the literature investigating the DWI features of AME, with the exception of a small series of three cases reported by Zhang et al. [11]. In their case series, ADC values above 1.5 × 10^−3^ mm^2^/s were obtained for three benign AME cases. In our study, lower ADC values were obtained, with a mean ADC value of 1.273 × 10^−3^ mm^2^/s. Recent studies with DWI have reported that an ADC threshold of 1.5 × 10^−3^ mm^2^/s or higher in BI‐RADS 4 lesions increases the likelihood of benignity and may reduce unnecessary biopsies [15, 16]. In this context, the ADC values in our cohort were below this cutoff, mimicking malignant features and supporting the necessity for biopsy. Considering the malignant potential of AME and the need for excision, these lower ADC values are clinically valuable, as they help ensure that such lesions are not overlooked.

The first‐line method for diagnosing AME is US‐guided core biopsy; however, achieving a definitive pathological diagnosis remains challenging due to the heterogeneous and complex nature of the tumor as well as the potential for inadequate sampling [7, 8, 12, 17]. According to the classification proposed by Rakha et al., AME represents a spectrum ranging from benign to atypical and malignant forms; therefore, limited sampling during core biopsy may not fully capture the architectural and cytological features required to accurately categorize the lesion within this spectrum [4]. From a radiological perspective, AME exhibits imaging features that overlap with various breast lesions. While some cases demonstrate benign characteristics such as circumscribed margins and hyperintensity on T2WI, others may present with increased vascularity, suspicious enhancement kinetics, and restricted diffusion on DWI, mimicking malignancy on CDUS and MRI [10]. This variability in imaging appearance may complicate radiologic interpretation. In our study, the presence of washout kinetic curves combined with relatively low ADC values further contributed to a malignant‐like imaging profile, potentially increasing the likelihood of radiologic–pathologic discordance and influencing clinical decision‐making. Notably, in our cohort, four out of 11 AME cases were initially discordant on core biopsy and were subsequently confirmed as benign AME following surgical excision. This finding underscores the potential for diagnostic discordance and the importance of careful radiologic–pathologic correlation, with surgical excision remaining essential for a definitive diagnosis.

There is a general consensus in the literature that wide excision is the preferred treatment for all AMEs to reduce the risk of local recurrence and to exclude malignancy, which may be difficult to differentiate with core biopsy alone [3–5, 7–14, 17]. Consistent with this, the relatively high rate of radiologic–pathologic discordance observed in our study further supports this approach, as definitive diagnosis and exclusion of atypical or malignant components often require evaluation of the entire lesion.

Our study has some limitations. First, we were only able to evaluate AME cases that were categorized as BI‐RADS 4 and above. It is possible that there were AME cases with lower BI‐RADS scores that remained stable under follow‐up without biopsy. Therefore, our study may be limited to the radiological features of AME cases detected due to biopsy prompted by suspicious characteristics. Second, our study was primarily focused on radiological findings and did not include a detailed evaluation of histopathological subtypes. Finally, owing to the rarity of this entity, the sample size was relatively small, which remains a common limitation in studies on AME.

In conclusion, although AME often demonstrates benign morphological characteristics in radiological evaluations, it is frequently assigned to a BI‐RADS 4 category on breast US and MRI due to its vascular and kinetic features that raise suspicion for malignancy. In our study, relatively low ADC values on DWI further supported this suspicious imaging profile. Considering the current treatment approach of wide local excision with negative surgical margins for lesions with malignant potential, the indication for pathological sampling based on these radiological findings is clinically crucial for ensuring accurate diagnosis and appropriate management.

**Nomenclature**
AME: Adenomyoepithelioma
BI‐RADS: Breast Imaging Reporting and Data System
US: Ultrasound
CDUS: Color Doppler ultrasound
MRI: Magnetic resonance imaging
DWI: Diffusion‐weighted imaging
ADC: Apparent diffusion coefficient
T2WI—T2: Weighted image

## Funding

This research received no specific grant from any funding agency in the public, commercial, or not‐for‐profit sectors.

## Disclosure

All authors have read and approved the final version of the manuscript. Pelin Seher Öztekin had full access to all of the data in this study and takes complete responsibility for the integrity of the data and the accuracy of the data analysis.

## Conflicts of Interest

The authors declare no conflicts of interest.

## Data Availability

The datasets generated and/or analyzed during the current study are not publicly available due to institutional and ethical restrictions but are available from the corresponding author on reasonable request.
